# *Lactobacillus plantarum* P2R3FA Isolated from Traditional Cereal-Based Fermented Food Increase Folate Status in Deficient Rats

**DOI:** 10.3390/nu11112819

**Published:** 2019-11-18

**Authors:** Aynadis Tamene, Kaleab Baye, Susanna Kariluoto, Minnamari Edelmann, Fabrice Bationo, Nicolas Leconte, Christèle Humblot

**Affiliations:** 1 Center for food science and nutrition, Addis Ababa University, Addis Ababa P.O. Box 150201, Ethiopia; aynadis.tamene@aau.edu.et (A.T.); kaleabbaye@gmail.com (K.B.); 2 Department of Food and Nutrition, University of Helsinki, P.O. Box 66, FIN-00014 Helsinki, Finland; susanna.kariluoto@helsinki.fi (S.K.); minnamari.edelmann@helsinki.fi (M.E.); 3 Département de Technologie Alimentaire, IRSAT, CNRST, Ouagadougou B.P. 7047, Burkina Faso; fabationo@gmail.com; 4 UMR Nutripass, IRD, University of Montpellier/Montpellier SupAgro, 34394 Montpellier, France; nicolas.leconte@ird.fr

**Keywords:** bioavailability, cereal, fermentation, folate, lactic acid bacteria, rats

## Abstract

Folate deficiencies are widespread around the world. Promoting consumption of folate-rich foods could be a sustainable option to alleviate this problem. However, these foods are not always available. Cereals, being a staple food, could contribute to folate intake. They are fermented prior to consumption in many African countries, and fermentation can modify the folate content. In Ethiopia, injera is a widely consumed fermented flat bread. The main drivers of its fermentation are lactic acid bacteria (LAB). The aim of this work was to isolate and identify folate-producing LAB from injera fermented dough and to evaluate their ability to increase folate status after depletion in a rat model. Among the 162 strains isolated from 60 different fermentations, 19 were able to grow on a folate-free culture medium and produced 1 to 43 µg/L (24 h, 30 °C incubation). The four highest folate producers belonged to the *Lactobacillus plantarum* species. The most productive strain was able to enhance folate status after depletion in a rat model, despite the relatively low folate content of the feed supplemented with the strain. Folate-producing *L. plantarum* strain has potential use as a commercial starter in injera production.

## 1. Introduction

Folate has a well-established role in preventing neural tube defects (NTDs) in the developing foetus [1,2]. NTDs are congenital malformations of the brain and spinal cord caused by failure of neural tube closure between 21 and 28 days post-conception [3]. Thus, sufficient folate is essential during early pregnancy when the embryo is growing rapidly and the folate requirement for DNA synthesis and methylation reactions is high. Insufficient or suboptimal intake of folate is also classically associated with megaloblastic anaemia [4]. 

Folic acid supplementation, food fortification with folic acid, and dietary diversification are currently used to address the problems associated with folate deficiencies [5]. Although it has been reported that consumption of the synthetic form of folate (folic acid) in supplementation and food fortification could prevent up to 75% of NTD [1], several studies have pointed to health concerns over the serious side effects of absorption of high amounts of folic acid [6,7]. Dietary diversification and promoting consumption of folate-rich foods could be sustainable options to alleviate the consequences of folate deficiency. However, depending on the season and on the geographic, agro-ecological, and socio-economic context, these foods are not always available [8]. In such cases, finding innovative sustainable ways of increasing the folate content in the diet is of great interest.

Cereals are staple-foods in many countries and contribute to folate intake, especially when consumed as whole grain [9]. In addition, in many African countries, cereal-based staple foods undergo a fermentation step with different microorganisms involved in the process, including lactic acid bacteria (LAB) [10]. Fermentation, in addition to improving the safety, shelf life, and sensory characteristics, can improve the nutritional value, and even give health-promoting properties to fermented foods [11]. For example, many authors suggest that fermented foods contain more folate than the original raw material [12,13]. As reviewed by Saubade et al. (2017), fermented cereal products could significantly increase daily folate intake in Africa [9]. Indeed, if many LAB can consume the folate initially present in the food matrix, several studies have shown that some can also synthesise folate during fermentation [9,14]. This is a strain-dependent trait, as it has been shown that different strains belonging to one species can synthesise or consume folate even when grown in the same conditions [9].

In Ethiopia, injera is a fermented flat bread widely consumed as a staple food [15]. It is often prepared from tef (*Eragrostis tef*), an ancient cereal crop indigenous to Ethiopia [16]. We recently showed that fermentation, one of the major processing steps in injera making, can increase the folate content of tef dough by up to 48% [17], suggesting that some microorganisms are able to synthesise folate. Given the observed variability of folate content in tef dough between traditional processing units, there is a need to identify the microorganisms responsible for folate production in tef dough during fermentation. 

Folate bioavailability is defined as “the fraction of the ingested nutrient that is available for utilisation in normal physiologic functions and for storage” [18]. It is usually accepted that the bioavailability of natural folate is lower than that of the synthetic folic acid used in food supplementation and fortification [19]. Nevertheless, a few authors have already demonstrated that folate-producing LAB are able to enhance folate status in rodent depletion–repletion assay and showed similar bioavailability to that of the synthetic folic acid [20,21]. 

The aim of the present study was to isolate and identify folate-producing LAB in the fermentation step in the preparation of tef injera. The strain producing the highest folate concentration in the culture medium was selected to evaluate its ability to restore normal folate status after depletion in a rat model. 

## 2. Material and Methods

### 2.1. Chemicals

Unless otherwise specified, all the chemicals used in this work were purchased from Sigma-Aldrich Chemie GmbH (Buchs, Switzerland). 

### 2.2. Sampling of Tef Dough

Samples were collected from 10 sub-cities of Addis Ababa, Ethiopia. In each sub-city, two households (where injera is traditionally prepared) were randomly selected. A total of 20 households were included in the survey. Detailed observations of the traditional injera making process in these households enabled the production of a standard flow diagram [17]. Sixty samples were collected from the 20 households after the first stage of fermentation (which lasts 3–4 days) on three separate occasions at an interval of approximately one month. Samples were collected from each household using a simple random sampling technique. The samples were collected aseptically and placed in sterile plastic bottles, covered with aluminium foil to protect them from direct contact with light, and transported to the laboratory in an ice box. Microbiological analysis was performed immediately on one aliquot of the dough sample for enumeration of presumptive LAB and for the isolation of folate-producing LAB. 

### 2.3. Enumeration of Presumptive LAB

Duplicate samples of tef dough (1 g) were homogenised with 9 mL of sterile saline solution (0.9% *w*/*v* NaCl) using a Stomacher 400 Lab Blender (Seward Medical, UK). The homogenates were serially diluted in saline solution, and 0.1 mL of subsequent serial 10-fold dilutions were spread plated on De Man, Rogosa, and Sharpe (MRS) agar (Merck, Schaffhausen, Switzerland) plates in duplicate. The plates were incubated under aerobic conditions at 30 °C for 48 h for the enumeration of presumptive LAB. 

### 2.4. Isolation of Potential Folate-Producing LAB

To maximise the chance of isolating folate-producing strains, the LAB were isolated on folate-free culture medium (Folic Acid Casei Medium (FACM), Difco, France). Briefly, 1 g of each dough sample was homogenised in 9 mL sterile saline solution (0.9% *w*/*v* NaCl). The homogenates were serially diluted and dilution of 10^−5^–10^−9^ were used to inoculate at 2% (*v/v*) sterile FACM in duplicate and then incubated under aerobic conditions at 30 °C for 48 h.

After incubation, media showing visible growth (10^−5^ and 10^−6^) were streak plated on sterile FACM plates and incubated at 30 °C for 72 h. Plates were prepared in duplicate for each dilution. A plate with the medium alone was left aside as a control to check for the absence of contamination.

After growth, single colonies with divergent morphology were picked and restreaked twice on fresh FACM medium to obtain pure cultures. After morphological examination (cell and colony morphology and Gram staining), all the bacterial strains were stored in FACM at −80 °C in glycerol (80%) until analysis of total folate and identification of the strains.

### 2.5. Total Bacterial Folate Analysis

Bacterial cell suspensions were cultured in 4 mL of FACM (1 % *v/v*) at 30 °C for 24 h. Total folate contents of the resulting cultures were determined using the microbiological method, after tri-enzyme extraction [22]. Analytical procedures were carried out under yellow or subdued light. Alternatively, aluminium foil was used to cover the samples and calibrants. Sample extracts were kept under nitrogen atmosphere.

#### 2.5.1. Extraction of Total Folate from Bacterial Cultures and Tri-Enzyme Treatment

To analyse total folate using the microbiological assay, the extraction procedure was adapted from Kariluoto et al. (2004). Samples (2 mL) were extracted in triplicate using the extraction buffer (pH 7.85; 50 mM CHES/50 mM HEPES, 10 mM 2-mercaptoethanol, sodium ascorbate 2% (*w*/*v*)), the tubes were flushed with nitrogen and heated at 100°C for 10 min. The samples were vortexed twice during thermal extraction and then cooled on ice. Extraction was followed by tri-enzyme treatment (α-amylase, Hog kidney conjugase, and protease) with some modifications: The pH was adjusted to 4.9 using HCl and α-amylase and Hog kidney conjugase were added to the extracted samples. Samples were flushed with nitrogen and incubated in a thermostatically-controlled water bath at 37 °C for 3 h under continuous agitation. The pH was readjusted to 7 using KOH, and protease was added to each sample extract, flushed with nitrogen, and incubated again (37 °C, 1 h, continuous agitation). After deactivating the enzymes in a boiling water bath and cooling with ice, the samples were brought to an exact volume of 10 mL with 0.5% sodium ascorbate (pH 6.1) and directly analysed using the microbiological assay. The same extraction procedure was applied to samples of non-inoculated FACM broth and to blank samples (extraction buffer plus the three enzymes), used as controls.

#### 2.5.2. Microbiological Assay

Ninety-six-well microtiter plates were used for the assay and the total folate content was determined based on the growth of folate-dependent *Lactobacillus rhamnosus* ATCC 7469 strain as the test organism, and 5-formyltetrahydrofolate (Merck Eprova AG, Schaffhausen, Switzerland) as the calibrant. Two dilutions were made from each sample extract using 0.5% sodium ascorbate solution and eight levels of calibrant (0–80 pg/well) in each plate. The plates were incubated at 36 °C for 18 h and turbidity was measured at 595 nm with a microplate reader (Multiskan EX; Labsystems, Helsinki, Finland). Method performance was confirmed by analysing a blank sample and certified reference material (BCR-121 wholemeal flour) in each set of samples. Folate contents within the certified value (500 ± 70 ng/g dry matter) were considered acceptable. In addition, folate contents of triplicate samples were not allowed to differ by ˃10%. 

Ninety-six-well microtiter plates were used for the assay and the total folate content was determined based on the growth of folate-dependent *Lactobacillus rhamnosus* ATCC 7469 strain as the test organism, and 5-formyltetrahydrofolate (Merck Eprova AG, Schaffhausen, Switzerland) as the calibrant. Two dilutions were made from each sample extract using 0.5% sodium ascorbate solution and eight levels of calibrant (0–80 pg/well) in each plate. The plates were incubated at 36 °C for 18 h and turbidity was measured at 595 nm with a microplate reader (Multiskan EX; Labsystems, Helsinki, Finland). Method performance was confirmed by analysing a blank sample and certified reference material (BCR-121 wholemeal flour) in each set of samples. Folate contents within the certified value (500 ± 70 ng/g dry matter) were considered acceptable. In addition, folate contents of triplicate samples were not allowed to differ by ˃10%. 

### 2.6. Identification of Isolated Folate-Producing LAB

Morphological observation was performed for all isolates of potential folate-producing LAB and genotypic identification was performed for the highest folate-producing isolates using sequencing of 16S rRNA coding genes following DNA extraction.

#### 2.6.1. DNA Extraction

The isolated LAB were routinely cultured in MRS broth at 30 °C overnight. The overnight bacterial cultures (2 mL) were centrifuged at 10,000× *g* for 10 min at 4 °C to pellet the bacteria. The final pellet was then washed one more time in 0.9 % (*w*/*v*) NaCl. DNA was extracted from the pellets using the Wizard genomic DNA purification kit (Promega, Charbonnières-les-Bains, France) with an additional lysis step using a bead beater (tissue lyser II, Qiagen, Les Ulis-Courtaboeuf, France) with zirconium beads (Biospect, Bartlesville, USA) as described by Turpin et al. (2011) [23]. First, the cells were lysed with 0.1-mm-diameter zirconium beads for 30 s, followed by 1 h of incubation at 37 °C in lysozyme (40 kU; Euromedex, France) and mutanolysin (10 U; Sigma, St Quentin Fallavier, France). Cell lysis was completed with Nuclei Lysis Solution (Promega, Charbonnières-les-Bains, France) according to the manufacturer’s instructions. RNA was removed with the RNase Solution (Promega, Charbonnières-les-Bains, France) and proteins with Protein Precipitation Solution (Promega, Charbonnières-les-Bains, France). DNA was precipitated with isopropanol and washed with 70% ethanol. DNA pellets were dried at 37 °C for 15 min in a SpeedVac (Thermo Fisher Scientific, Villebon-sur-Yvette, France). The pellets were then re-suspended with 200 µL of rehydration solution at 4 °C overnight under agitation. The concentration, purity, and quality of the DNA were confirmed by measuring absorbance at 230, 260, and 280 nm (NanoVueTM, GE Healthcare, Sweden) and then separated on agarose gel, followed by staining with ethidium bromide. 

#### 2.6.2. 16S rRNA Gene Sequencing

For 16S rRNA gene sequencing, primers W001 [24,25] and 23S1 (GenBank accession no. J01695) were used to amplify the 16S rRNA gene, including the intergenic region located between 16S rRNA and 23S rRNA. PCR products were sequenced by GENEWIZ (Takley-Essex, UK) using the primers SP3, SP4, and SP5 [25,26]. Gene sequences were aligned using ClustalW to generate single consensus sequences. Each sequence was identified by comparing it with sequences from the Ribosomal Database Project 11 (RDP11) (http://rdp.cme.msu.edu) and >97% similarity was accepted. 

### 2.7. Bioavailability of Folate Produced by the Selected Strain

The most efficient folate-producing strain (*L. plantarum* P2R3FA) obtained from tef fermented dough was lyophilised and added to a folic acid-deficient rat diet (FADD; Cat. N° 517812, Dyets, Bethlehem, PA, USA) to evaluate the bioavailability of folate produced by the selected strain. Briefly, the selected LAB strain was grown in MRS agar medium at 30 °C for 24 h. A colony was picked from each pure culture plate, grown successively in MRS broth. Several batches of fermentations were performed to obtain the desired number of cells with intracellularly accumulated folate. Cells were harvested by centrifugation at 14,000× *g* for 7 min. The pellet was washed in a sterile saline solution (0.9% *w*/*v* NaCl). Subsequently, cells were freeze-dried in the dark to prevent folate loss and were homogenously mixed with the FADD. The folate contents of the freeze-dried cells were quantified using the microbiological assay method described in Section 2.5.2.

#### 2.7.1. Rat Depletion–Repletion Assay

All the experiments were performed in accordance with the animal care guidelines of the Guide for the Care and Use of Laboratory Animals published by the National Research Council of National Academics (2011) [27]. All procedures were approved by Addis Ababa University Ethical Clearance Review Board. After weaning, thirty 3-week-old male Wistar rats, initial weight 316 ± 23 g, were obtained from the Ethiopian Public Health Institute and were singly housed in stainless steel plastic covered cages. 

**Figure 1:**
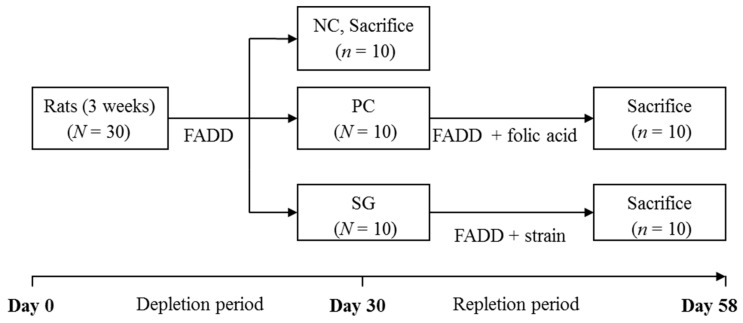
Folate depletion–repletion feeding protocol. NC: Negative control group, rats fed with a folic acid-deficient diet (FADD); PC: Positive control group, rats fed FADD containing 2 mg folic acid/kg of diet; SG: Strain group, rats fed with FADD supplemented with lyophilised *L. plantarum* P2R3FA strain containing 0.25 mg of folate/kg of diet. *N* = total number of animals per group. *n* = number of animals used for folate analysis.

Animals remained under controlled environmental conditions (temperature 22 ± 2 °C, humidity 55 ± 2%) with 12 h light/dark cycles with access to food and water ad libitum throughout the study. Certified FADD (Cat. N° 517812, Dyets, Bethlehem, PA, USA) and FADD with 2 mg of folic acid per kg of diet (Cat. N° 517802, Dyets) were used in this study. The overall experimental protocol is summarised in Figure 1. 

The rats were randomly selected for the study and were fed with FADD (negative control diet) for 30 days (depletion period). After the depletion period, 10 rats (negative control group) were randomly selected and sacrificed for red blood cell and serum folate analysis to confirm the deficiency. 

The 20 remaining rats were divided into two experimental groups, each containing 10 rats with equal mean weights (depletion–repletion groups). The first group (positive control group) of rats was fed with the FADD with 2 mg of folic acid per kilogram of diet (positive control diet) and the other group (strain group) of rats was fed with FADD supplemented with the lyophilised strains with 0.25 mg of folate per kilogram of diet (experimental diet) for 28 days (repletion period). 

Animal growth (weight) and food consumption were recorded during the depletion and repletion periods. After the repletion period, all the rats in each group were sacrificed for red blood cell and serum folate analysis.

#### 2.7.2. Collection of Blood Samples

Animals were anaesthetised with an intraperitoneal injection of 3.0 mL of ketamine (10% *w*/*v*)–xylazine (2% *w*/*v*; 40:60 *v/v*, Alfasan, Woerden, The Netherlands) per kilogram of animal weight and bled by cardiac puncture. Blood was transferred into tubes with or without anticoagulant, depending on the objective of the analysis.

For serum samples, blood without anticoagulant was allowed to clot and serum was separated by centrifugation at 1500× *g* for 10 min. For preparation of whole-blood samples for erythrocyte folate analysis, an aliquot of blood containing anticoagulant was mixed with 1 mL of 1% ascorbic acid solution. The solution was then thoroughly mixed by inverting the sample tubes 10 times to lyse the red blood cells. Folate analysis of all samples was performed using microbiological assay as described in Section 2.5.2 but without the tri-enzyme treatment.

### 2.8. Statistical Analysis

Statistical analysis of folate of the isolated strains, folate of serum and erythrocytes, total feed and folate intake and weight gained by the experimental rats were computed using SPSS version 20. The folate analyses were carried out in triplicate and the average values and standard deviations were calculated. Differences between means of folate values were evaluated using one-way analysis of variance (ANOVA) and Tukey’s post hoc test. Differences in means were considered statistically significant with a *p*-value ≤ 0.05.

## 3. Results

### 3.1. Enumeration of Presumptive LAB from Tef Fermentation

**Figure 2:**
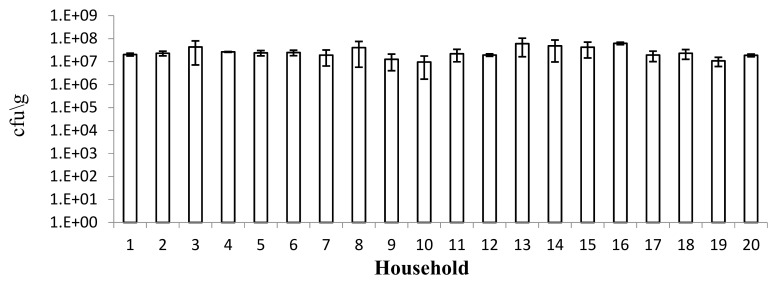
Counts of presumptive lactic acid bacteria (LAB) at the end of tef fermentation. Bars indicate standard deviations among three samples collected from each of the 20 households. There were no statistical differences between the households (*p* < 0.05).

The total count of presumptive LAB ranged from 3.8 × 10^6^ to 1.5 × 10^8^ with an average of 2.5 × 10^7^ colony forming units per gram (cfu/g) of fermented dough (Figure 2). No significant differences were observed (*p* ˃ 0.05).

### 3.2. Isolation and Identification of Folate-Producing LAB

**Figure 3:**
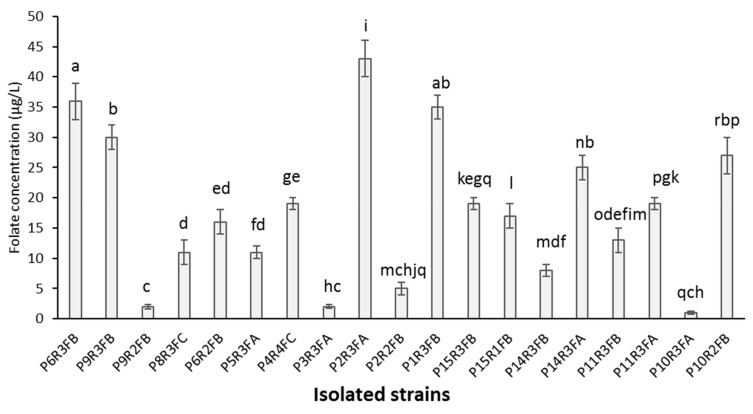
Production of folate by 19 LAB isolated from tef dough when incubated at 30 °C for 24 h in Folic Acid Casei Medium (FACM). Bars indicate standard deviations among analytical replicates. Mean values with different superscript letters indicate a statistically significant difference between experimental groups (*p* < 0.05).

A total of 162 potential folate-producing LAB strains were isolated from the 60 different tef fermentations. Phenotypic characterisation of all the 162 isolates was performed and the results revealed all of them to be rod-shaped Gram-positive bacteria. Among the 162 isolated strains, 19 were able to grow on FACM after 24 h of incubation at 30 °C. After the 24-h incubation period, total folate was assessed using the microbiological assay and ranged from 1 to 43 µg/L with high individual variability (Figure 3). The four highest folate-producing isolates of bacteria, P1R3FB, P2R3FA, P6R3FB, and P9R3FB, were selected for the purpose of identification. The result of 16S rRNA coding gene sequencing revealed that all four isolates shared 98–100% sequence identity with *L. plantarum.*

### 3.3. Rat Depletion–Repletion Assay

Considering the experimental design, the rats in the negative control group were sacrificed 28 days earlier than the rats in the two experimental groups. As expected, the total feed intake and the weight gained by the negative control group were significantly lower than in the two experimental groups. Feed intake between the two experimental groups fed with folic acid and the strain was similar (699 ± 65 g and 700 ± 86 g, respectively), as well as weight gain (66 ± 19 g and 70 ± 19 g, respectively, Table 1). The calculated total folate intake (1398 ± 129 µg) by the positive control group, which consumed 2000 µg of folic acid per kilogram of diet, was significantly higher than the total folate intake (175 ± 21 µg, *p* ˂ 0.05) of the strain group that consumed 250 µg of folate per kilogram of diet (Table 1).

**Figure 4:**
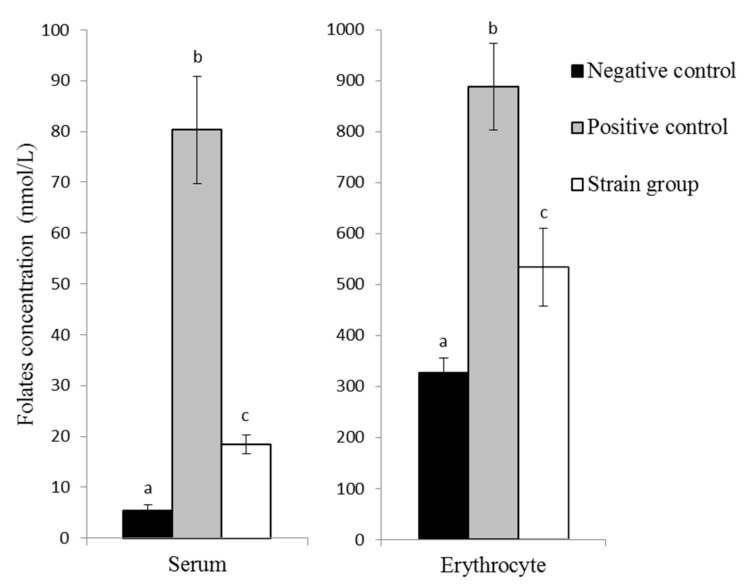
Folate concentrations determined in serum and erythrocytes after folate depletion–repletion periods. Negative control: Rats fed with FADD for 30 days; positive control: Rats fed a FADD for 30 days, followed by 28 days of feeding with a FADD supplemented with 2 mg of folic acid/kg; strain group: Rats fed a FADD for 30 days, followed by 28 days of feeding with FADD supplemented with *L. plantarum* P2R3FA strain containing 0.25 mg of folate/kg of diet. Values are means ± standard deviations. Mean values with different superscript letters indicate a statistically significant difference between experimental groups (*p* < 0.05).

Mean erythrocytes and serum folate concentrations in the three groups of rats differed significantly (*p* ˂ 0.05; Figure 4). The depletion period was successful, since the erythrocytes and serum folates of all depleted rats were found to be very low.

Both groups of repleted rats (positive control and strain groups) exhibited higher erythrocyte and serum folate levels than the depleted rats. The concentration of folate in erythrocytes was significantly higher in the positive control group (888 ± 86 nmol/L) than in the strain group (534 ± 29 nmol/L). The same was true for serum samples, where a significantly higher folate concentration was found in the positive control group (80 ± 10 nmol/L) than in the strain group (18 ± 2 nmol/L). 

## 4. Discussion

Since the few literature sources on microbial investigation of fermented tef-injera dough showed the predominance of LAB in the fermentation process, we chose to focus on LAB in the present work. In this study, the presence of a high number of presumptive LAB in tef-*injera* dough was confirmed and was similar in all the samples collected in the 20 households. Indeed, although small numbers of *Enterococci*, yeast, and mould species have been reported, LAB have been shown previously to be predominant [28,29,30]. 

Among the 162 strains isolated from the 60 different tef fermentations, 19 were effectively able to produce folate, from 1 to 43 µg/L. It is generally considered that almost all LAB are unable to produce folate. Indeed, some genes coding enzymes involved in folate biosynthesis or in the precursors synthesis, such as p-Aminobenzoic acid, are lacking [14]. Nevertheless, since some specific folate-producing LAB have been isolated and successfully used to maximise folate production during fermentation of food products, interest in this topic has increased significantly recently. But most of the studies have focussed on dairy products, and only a few studies have been conducted on cereal-based fermented products, as reviewed by [9]. Although it is often difficult to compare data on total folate content of LAB with the existing literature, due to differences in the methods and in the media used in different studies, the total folate concentrations obtained in the present study are in the same range as previously reported folate concentrations (up to 148 µg/L) produced by LAB isolated from different sources and grown in the same folate-free culture medium FACM [9]. 

The highest folate producer among our isolates (*L. plantarum* P2R3FA) was able to grow and produce 43 µg/L of total folate in FACM. The bioavailability of natural folate is often considered to be lower than that of synthetic folic acid [19,31]. The bioavailability of folate produced by this strain was assessed using a rat depletion–repletion assay. Drastic serum and erythrocyte folate concentration decreases were observed in rats fed with FADD during the 30-day depletion period and justified the use of this model to study folate repletion. Both these deficiencies were reversible, as confirmed by the serum and erythrocytes folate concentration increases in the two groups of rats that received either folic acid or the lyophilised strain during the 28-day repletion period. 

Even though the amount of folate consumed by the strain group (175 ± 21.5 µg) was far lower than the total folate consumed by the positive control group (1398 ± 129.2 µg), the strain was able to increase the folate status of depleted rats. The clear response of *L. plantarum* administration in the folate-deficient diet observed in the folate concentration in serum and erythrocytes indicates that bacterial folate was available for absorption in the gastrointestinal tract of rats. Interest in administrating food grade folate-producing bacteria to improve folate status has been increasing and some authors have reported that the serum, erythrocyte, and liver folate concentration can be increased [20,21]. For example, the administration of different freeze-dried strains from *Bifidobacterium* genus to rats allowed an increase in serum folate concentration form 4.8 to 9.1 nmol/L after two weeks. In our case, the increase was even more pronounced, since we reached 18.4 nmol/L after one month of feeding with *L. plantarum* P2R3FA administration [20]. Another study used *Lactococcus lactis* genetically-modified to synthesise folates with different polyglutamate tail length. The depletion phase lead to significant decrease in folate concentration in liver, kidney, and serum, but not in erythrocytes. But these authors observed that the low amount of folate from *Lactococcus lactis* strains (250 µg/kg of diet) was able to compensate for folate depletion and showed similar bioavailability in terms of increasing serum folate concentrations in animals that received folate-producing strains compared with the same amount of commercial folic acid. The different polyglutamyl tail lengths did not affect the folate bioavailability [21]. Given the serious health and environmental concerns linked to the use of genetically modified organisms, in the present study we used a completely wild LAB strain combined with normal diet, and our results are in good agreement with the previous findings [20,21]. 

Since cereal-based fermented foods are widely consumed in Africa, using folate-producing strains as starter cultures during fermentation of foods could be an efficient way of enhancing folate status. But folate is also a highly sensitive molecule, since a substantial amount of the initial folate can be lost during preparation or storage of the food [32]. Hence, the oral administration of a pure strain able to synthesise folate is a possible alternative and/or additional way than eating folate-rich foods to fight folate deficiencies. Therefore, we highly recommend further studies on the use of *L. plantarum* strain P2R3FA as a pure strain or as a starter culture to produce folate-enriched foods.

## Figures and Tables

**Table 1:** Feed and folate intakes and body weight gain in rats after the depletion–repletion assay.

Indices	Depletion	Repletion	
	Negative Control Group	Positive Control Group	Strain Group
**Total feed intake (g)**	660 ± 79 ^a^	699 ± 65 ^b^	700 ± 86 ^b^
**Weight gained (g)**	54 ± 20 ^a^	66 ± 19 ^b^	70 ± 19 ^b^
**Total folate intake (µg)**	0	1398 ± 129^b^	175 ± 21 ^a^

Negative control group: Rats fed with FADD for 30 days; positive control group: Rats fed a FADD for 30 days, followed by 28 days of feeding with a FADD supplemented with 2 mg of folic acid/kg; strain group: Rats fed a FADD for 30 days, followed by 28 days of feeding with FADD supplemented with *L. plantarum* P2R3FA strain containing 0.25 mg of folate/kg of diet. Values are means ± standard deviations. Mean values across the treatment groups with different superscript letters are significantly different (*p* < 0.05).
